# Survival, rarity, and extinction in tropical stony corals

**DOI:** 10.1111/cobi.70200

**Published:** 2025-12-27

**Authors:** Bryan Wilson, Peter J. Edmunds

**Affiliations:** ^1^ John Krebs Field Station, Department of Biology University of Oxford Wytham UK; ^2^ Department of Biology California State University, Northridge Northridge California USA

**Keywords:** *Acropora cervicornis*, *Acropora palmata*, *Ctenella chagius*, *Dendrogyra cylindrus*, rarity, reef‐building corals, corales formadores de arrecifes, rareza, *Ctenella chagius*, *Dendrogyra cylindrus*, *Acropora cervicornis*, *Acropora palmata*, 造礁石珊瑚, 稀有性, Ctenella chagius, Dendrogyra cylindrus, Acropora palmata, Acropora cervicornis

## Abstract

Many reef‐building tropical corals are becoming rare. We considered the meaning of rarity in corals and highlighted taxa that have reached low abundances in the last few decades. The difficulties of quantifying rarity in the marine environment arise from the sheer scale and 3‐dimensional nature of the biome and the inherent challenges therein of ecological surveys with scuba. To meet the demands of coral conservation biology in the 21st century, we suggest that contemporary studies of coral communities will require enhanced capacity to identify species and a species‐specific focus on corals occurring at low abundances, which traditional ecological approaches to quantifying populations of benthic marine organisms have a limited capacity to address. Now is the time to revise scientific approaches to respond to the challenges posed by the need to understand and protect rare tropical corals.

## Abstract (es)

Supervivencia, rareza y extinción de los corales rocosos tropicales

**Resumen:**

Muchos corales tropicales formadores de arrecifes se están volviendo raros. Consideramos el significado de la rareza en los corales y destacamos los taxones que han bajado en abundancia en las últimas décadas. Las dificultades para cuantificar la rareza en el medio marino se derivan de la enorme escala y la naturaleza tridimensional del bioma, así como de los retos inherentes a los estudios ecológicos con buceo. Para satisfacer las exigencias de la biología de la conservación de los corales en el siglo XXI, sugerimos que los estudios contemporáneos de las comunidades de corales requieran una mayor capacidad para identificar especies y un enfoque específico en los corales que se encuentran en bajas abundancias, algo que los enfoques ecológicos tradicionales para cuantificar las poblaciones de organismos marinos bentónicos tienen una capacidad limitada para abordar. Ahora es el momento de revisar los enfoques científicos para responder a los retos que plantea la necesidad de comprender y proteger los corales tropicales raros.

## 热带石珊瑚的生存、稀有性与灭绝

**【摘要】** 许多热带造礁石珊瑚正变得越来越稀少。本文探讨了珊瑚稀有性的意义, 并重点指出了近几十年来数量锐减的类群。在海洋环境中量化稀有性面临许多困难, 这是由于珊瑚的广泛分布及其三维特性, 以及通过水肺潜水进行生态调查的固有挑战。为满足21世纪珊瑚保护生物学的需求, 我们提出, 当前的珊瑚群落研究需提升物种鉴定能力, 并关注低丰度珊瑚的物种特异性研究, 而量化底栖海洋生物种群数量的传统生态学方法在这方面存在局限性。目前亟需改进科学方法, 以应对理解和保护稀有热带珊瑚所面临的挑战。**【翻译:胡怡思;审校:聂永刚】**

## INTRODUCTION

Global climate change and other human disturbances have left a fingerprint of perturbed ecology on almost every biome (Hannah et al., 1995; Williams et al., 2020) and created novel communities relative to those of recent centuries (Hobbs et al., 2009; Radeloff et al., 2015). The changes are diverse and pervasive, but those leading to large increases in abundance of uncommon or invasive taxa (Côté & Smith, 2018; Langin, 2018) or to large declines in the abundance of common or functionally significant organisms (Cramer et al., 2020; Lessios et al., 1984) have attracted great attention. To evaluate these trends in the marine biome, ecologists must modify survey methods to effectively quantify rare organisms that are likely to face elevated risks of extinction.

Tropical coral reefs are threatened by climate change (Bellwood et al., 2004), with bleaching playing a prominent role in driving declines in coral abundance (Heron et al., 2016; Tebbett et al., 2023). An update in 2024 to the 2008 International Union for the Conservation of Nature (IUCN) Red List assessment of Atlantic corals has elevated the extinction risk of half of the species (Gutierrez et al., 2024). Together with a variety of other disturbances (Hoegh‐Guldberg et al., 2007; Hughes, Barnes, et al., 2017), these events have been associated with reduced abundances of many corals (Hughes et al., 2018; Toth et al., 2019). Although multidecadal surveys of reefs have highlighted long‐term trends in coral cover (De'ath et al., 2012; Edmunds, 2024), less is known about the abundances of individual species (Dietzel et al., 2021). Some corals have been described as rare (DeVantier & Turak, 2017), or threatened with extinction (Carpenter et al., 2008), and one species (the hydrocoral *Millepora boschmai*) was declared extinct in the Galapagos in 1991 (Glynn & de Weert, 1991) but rediscovered soon after (Glynn & Feingold, 1992).

Motivated by these trends, scientists have investigated the causes of widespread coral mortality and proposed actions to reduce the rate and extent of population decline (Hughes, Kerry, et al., 2017; Tebbett et al., 2023). Research has expanded from traditional ecology approaches to consider managing coral survival through “oasis” sites (Guest et al., 2018) and “bright spots” (Cinner et al., 2016), as well as the treatment of select species as endangered (Brainard et al., 2011). For endangered species, their survival might be elevated through captive breeding (Banaszak et al., 2023; Zoccola et al., 2020), restoration (Suggett et al., 2024), colony translocation (Hoegh‐Guldberg et al., 2008), assisted migration (Dela Cruz & Harrison, 2020), marine protected areas (MPAs) (Mellin et al., 2016), improvements in seawater quality (MacNeil et al., 2019), and fisheries management (Weijerman et al., 2018). Although these approaches would probably be beneficial, the most important contribution to the survival of rare corals remains the reversal of climate change and net removal of atmospheric carbon dioxide (Hoegh‐Guldberg et al., 2007; Zickfeld et al., 2013). Nevertheless, attention afforded to the coral reef crisis (Hughes, Barnes, et al., 2017) has not fully embraced the demographic implications of low abundances of coral species. Declines in abundance, culminating in rarity (Gaston, 1997), have important implications, and it is timely to revise ecological approaches to better quantify low‐abundance corals (DeVantier & Turak, 2017).

In writing this paper, the approaches we have taken are contextualized by evidence that multiple corals have transitioned from high to low abundance (Hughes et al., 2018; Neely et al., 2021). We contrasted the meaning of *colloquial rarity* with *scientific rarity* (terms defined below) and considered the conditions under which rarity might lead to extinction for corals. We considered that low abundances for many corals is a relatively new phenomenon for contemporary marine ecology and describe the challenges such low abundance poses for traditional population survey techniques. We also considered the research required to determine the causes and consequences of rarity for reef corals.

## THE MEANING OF *RARE*

Rarity is an elusive concept and is primarily defined by shifts in relative abundance and distributional ranges (Gaston, 1997). Colloquially, a species needs only to change from being perceived as common to being deemed rare within a finite area or to have a discernible lower abundance than a dominant taxon to be described as rare. Scientifically, a species is considered rare based on multiple factors (Gaston, 1997). Rabinowitz (1981) detailed 7 forms of rarity through binary combinations of dichotomized distributional range, habitat tolerance, and population size. Gaston (1997) suggested rarity could be visualized in a 3‐dimensional space with axes defined by relative weightings of biology (history and abundance), threats (risk of extinction), and value (ecological importance). Elucidating the biological meaning of rarity remains challenging, for rarity defined by low abundance is a poor predictor of extinction (Harnik et al., 2012), and holistic rarity (Gaston, 1997) does not guarantee extinction (Harnik et al., 2012). In part, this is because some species have been rare throughout evolutionary time and may be adapted to this state (the “intrinsic‐trait hypothesis” [Harrison et al., 2008]). Other species that have quickly become rare may lack the traits necessary to persist at low population densities. Harrison et al. (2008) hypothesized several alternative explanations for the persistence of naturally rare species, including the “favorable‐environment hypothesis” (favorable environmental conditions, rather than intrinsic biological traits, predict rare species) and the “rapid‐speciation hypothesis” (which states that rare species die more quickly than abundant species and are more likely to rapidly speciate). *Rarity* assumes a special meaning as a prelude to extinction (Brook et al., 2008; Mace & Lande, 1991), although species that have been rare for millennia are less likely to become extinct (Hambler & Canney, 2013). The fossil record provides insight into the fate of rare species (Hull et al., 2015; Raja et al., 2021) and shows that distributional range is the best predictor of extinction (Budd & Pandolfi, 2010).

Most species in a community are rare (Preston, 1948), and population surveys generate a J‐shaped histogram for abundances arrayed from low to high (McGill et al., 2007). *Rare* is often a subjective term to describe organisms that have become uncommon, usually relative to adjacent organisms that are taxonomically similar (Gaston, 1997). There is no benchmark for abundance‐based rarity, either in the proportional reduction in population size required to attain rarity or the spatial extent over which the reduction applies (Gaston, 1997). Many examples of abundance‐based rarity are cases of local low abundance, unless they can be augmented by information on other aspects of organismal biology, ecological value, or the threats to which the species are exposed. Rarity often does not have a demographic foundation, and it is impossible to know how abundance translates into the rate at which populations shrink or to evaluate the projected time to extirpation or extinction. It may therefore be unreliable to classify corals as rare for conservation purposes because they occur at low local abundance and occupy a limited geographic range or restricted niche or because the effort to find them on the reef has not been comprehensive.

The IUCN Red List uses particular criteria to categorize the likelihood of extinction of taxa (Mace et al., 2008), and there is no alternative global conservation mechanism as independent or as scientifically robust (Betts et al., 2020). With the application of quantitative rules, which relate to population size, range area, and rate of population decline, species are qualitatively assigned to categories of extinction risk (Collen et al., 2016). The IUCN Red List includes 166,061 species (IUCN, 2024); however, there is less threat status data for marine than nonmarine taxa (Webb & Mindel, 2015), and invertebrates are historically underrepresented (Cardoso et al., 2012; Eisenhauer et al., 2019). Although vertebrates and invertebrates are broadly assessed in similar proportions under each of the 5 IUCN criteria, cnidarians are more frequently designated under criterion A (population reduction), which highlights species at greatest risk due to a steep rate of decline in population size, regardless of whether they are currently abundant or rare (Collen et al., 2016). A meta‐analysis of the distribution of Indo‐Pacific corals (Dietzel et al., 2021) showed that there was overlap in the population sizes of coral species assigned to different IUCN categories and that species at high risk had smaller population sizes than corals at low risk of extinction. However, the limited geographic scope of the study precludes “robust conclusions about global extinction risk” (Muir et al., 2022). It has also been suggested that the criteria used to assess the conservation status of corals should identify the life‐history traits that are positively associated with extinction risk of corals and ultimately must be directed at species that can be definitively identified (Bridge et al., 2020). Given these discrepancies and the number of coral species for which IUCN classifications are data deficient, a major revision of threat status criteria is needed for corals (Dietzel et al., 2021).

Aligning much of the data used to prepare IUCN Red List entries with measures of phylogenetic diversity (Faith, 1992), the Zoological Society of London's EDGE of Existence programme uses the EDGE (evolutionarily distinct and globally endangered) metric (Isaac et al., 2007) to rank threatened species by their risk of extinction. Because the implications of rarity are most acute for evolutionarily distinct corals, for which their loss would have a disproportionately large effect on scleractinian diversity (Huang, 2012), the EDGE metric extends beyond IUCN's Red List criteria because it represents a more comprehensive means to support targeted conservation management of reef corals.

Despite the advanced state of development of conceptual frameworks to evaluate rarity in terrestrial environments, their application in the marine realm remains limited. In the oceans, rarity must be evaluated in an environment in which surveys are completed from boats or through underwater surveys with scuba or autonomous vehicles. Since the advent of scuba, the summed area of benthic surveys on coral reefs amounts to <0.001% of the habitat, and this area does not include the ranges of many corals (DeVantier & Turak, 2017). Repeated surveys of a single site can quantify coral abundance over time and provide early detection of low abundance and ongoing declines in population size. The high spatial variation in coral communities (Williams et al., 2015) indicates that single‐site surveys are likely to generate misleading indications of coral rarity.

Coral reefs at >30 m depth, categorized as mesophotic (to 150 m) (Kahng et al., 2010), provide an interesting challenge to quantifying coral abundance. The need to access these areas has increased since the deep reef refuge hypothesis (DRRH) (Bongaerts et al., 2010; Laverick et al., 2018; Semmler et al., 2017) motivated the search for rare corals in deeper water. Although support for this hypothesis remains limited (Bongaerts & Smith, 2019), it highlights the need to evaluate the abundance of corals across their full depth range to evaluate their candidacy for consideration as rare.

Claims that select coral species have become rare based on data originating from the use of traditional ecological techniques are prone to categorizing corals as pseudorare (“type I” rarity) (Blackburn & Gaston, 1997), and these species could be categorized as common if they persist at high abundance at a relict site (“type II” rarity) (Blackburn & Gaston, 1997). In addition to the logistical challenges of conducting underwater surveys, the biology of a taxon obscures the meaning of rarity. Corals are surrounded by seawater that can transport their pelagic propagules over large distances (Burt et al., 2024; Graham et al., 2008) dependent on a range of biophysical processes (Cowen & Sponagule, 2009; Edmunds et al., 2018). This allows discrete populations to operate as part of larger metapopulations (Botsford et al., 2009), such that populations at low abundances have the potential to benefit from subsidies from populations at high abundances (Spiecker et al., 2016). However, corals with restricted larval dispersal may be unable to recolonize habitat lost after disturbances, especially on remote reefs (Dietzel et al., 2021). Although long‐distance larval dispersal to remote locations can occur (Burt et al., 2024), it is likely to be rare on ecologically relevant timescales. Nevertheless, the key question is often not whether populations are connected by larval transport, but over what scales of space and time are these effects expressed? Recent data suggest that coral community recovery following disturbances depends on local sites acting as either sources or sinks for larval recruitment (Ani et al., 2024; Edmunds et al., 2018). As coral population sizes decline and experience asynchronous reproduction or low per capita reproductive outputs (Knowlton, 2001; Shlesinger & Loya, 2019), separation among conspecific colonies is likely to become so great as to render chances of cross fertilization low (Mumby et al., 2024), favoring the development of Allee effects (Courchamp et al., 1999).

**FIGURE 1:**
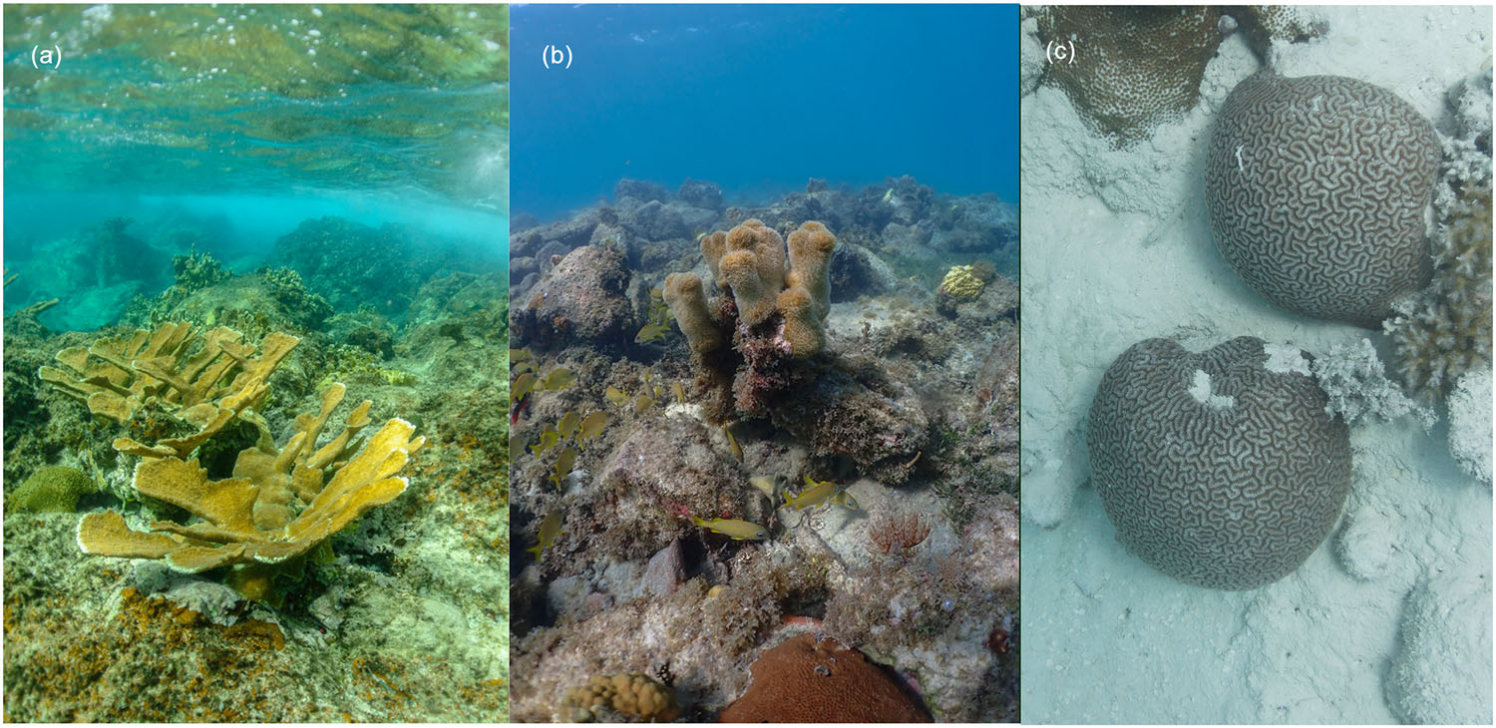
Reef‐building corals that now are so uncommon on shallow reefs that they are candidates for being described as rare: (a) *Acropora palmata* in St. John, U.S. Virgin Islands (August 2022), (b) one of the last *Dendrogyra cylindrus* in Great Lameshur Bay, St. John (November 2023), and (c) *Ctenella chagius* in Middle Brother Lagoon, Chagos Archipelago (July 2023).

A defining feature of many coral communities is that most species occur at low abundances (Dornelas et al., 2006) and some are categorized as rare, the result of which is that the summed percentage cover of corals typically is dominated by a few species. A meta‐analysis of coral cover and species abundances from 1997 to 2006 at >900 Indo‐Pacific sites revealed that *Acropora*, *Porites*, and *Favia* comprised 75% of species (Dietzel et al., 2021). These genera were characterized by large geographic ranges and high local abundance, and they dominated benthic space proportionate to their relative abundance (Dietzel et al., 2021). On a shallow (i.e., <15 m depth) Caribbean reef in the 1950s (Goreau, 1959), a dominating canopy of *Acropora palmata* or a framework of *Orbicella annularis* would have been populated at low abundances by other corals, some of which likely had conspecific colonies separated on a decimeter scale. Most biologists who have worked on coral reefs for decades have witnessed numerous common coral species transition to low abundances, and many of these biologists might describe such species as rare. We considered 3 examples (Figure 1) of what rare can mean for corals on present‐day reefs.

## *Ctenella chagius* IN THE INDIAN OCEAN

*Ctenella* may be the world's most endangered coral (Huang, 2012) (a monotypic genus represented by *C. chagius* Matthai [Matthai, 1928]). It is considered endemic to the Chagos Archipelago in the Central Indian Ocean (Sheppard et al., 1983), which serves as a putative “centre of abundance” for the species (DeVantier & Turak, 2017). *Ctenella* was found throughout the Western Indian Ocean; the Percy Sladen Trust Expedition in 1905 collected colonies from the Saya de Malha bank between Mauritius and the Seychelles. A few *Ctenella* were found in the shallow sea grass meadows of Saint‐Brandon (Mauritius) in 2010 (Obura, 2012), but none have been reported since.

From benthic surveys carried out during the 1978–1979 UK Joint Services Chagos Research Expedition, *Ctenella* was recorded as one of the 25 most common corals (at <45 m depth) in the Chagos Archipelago (Sheppard et al., 1983), which has been a no‐take MPA since 2010. However, its abundance has declined drastically (Sheppard et al., 2017), and <150 colonies have been found in the last 5 years (Wilson et al., 2024). Of these, 101 were in a shallow lagoon (<1 km^2^) at Middle Brother Island on the edge of the Great Chagos Bank (Wilson et al., 2024). Consequently, the IUCN Red List category for *Ctenella* was amended in 2024 from endangered to critically endangered (Wilson et al., 2024); it is also listed as an EDGE species (http://www.edgeofexistence.org/species/ctenella‐chagius/). The primary cause of this decline in population size is thought to be seawater warming (Sheppard & Sheppard, 2019).

## *Dendrogyra cylindrus* IN THE CARIBBEAN

*Dendrogyra cylindrus* is an iconic Caribbean coral growing as pillar‐shaped colonies several meters high (Cavada‐Blanco et al., 2020; Marhaver et al., 2015), often with multiple columns up to ∼13‐cm diameter (Human & DeLoach, 2013). Similar to *Ctenella*, *Dendrogyra* is a monotypic genus and an EDGE species (http://www.edgeofexistence.org/species/pillar‐coral/). Although it has not been common on Caribbean reefs since quantitative ecology began in the 1960s, over much of the last 70 years it has been conspicuous (Chan et al., 2019; Jones et al., 2021; Marhaver et al., 2015), often in high‐wave‐energy locations. Nevertheless, the low abundance of *Dendrogyra* has placed it in the colloquial rare category (Marhaver et al., 2015; Neely et al., 2018). In 2014, stony coral tissue loss disease started to kill corals throughout the Caribbean (Precht et al., 2016), and *Dendrogyra* has been among the most susceptible to this disease (Neely et al., 2021). By 2022, live *Dendrogyra* were rare throughout Florida's Coral Reef (Neely et al., 2021) and the wider Caribbean (Cavada‐Blanco et al., 2022), leading to initiatives to list it as critically endangered (Cavada‐Blanco et al., 2022).

## *Acropora* spp. IN THE CARIBBEAN

*Acropora palmata* and *A. cervicornis* were among the most important reef‐building corals in the Caribbean prior to the 1980s (Bruckner, 2002; Goreau, 1959), and this was true for at least ∼250,000 years (Jackson, 1992; Pandolfi & Jackson, 2006). On shallow fore reefs, the “palmata zone” created a framework of arborescent colonies up to 4 m in diameter (Acropora Biological Review Team, 2005). Below this canopy, other reef‐building corals persisted in an understory habitat (Porter & Meier, 1992) characterized by reduced light, flow, and space, relative to the open reef. Slightly deeper was the “*cervicornis* zone” that was a complex 3‐dimensional framework of stout branches (Rylaarsdam, 1983). *Acropora* throughout the region were severely affected by white band disease in the early 1980s and transitioned to uncommon within a few years (Aronson & Precht, 2001).

Over the following decades, Caribbean *Acropora* have been negatively affected by multiple disturbances (Aronson & Precht, 2001; Cramer et al., 2021; Woodley et al., 1981), so that by the new millennium they were listed as threatened (NOAA, 2006). *Acropora* spp. have been uncommon on most Caribbean reefs for decades (Jackson et al., 2014; Steneck et al., 2019), although they can still be found in some locations, including the U.S. Virgin Islands (Edmunds, 2014; Muller et al., 2014), the north shore of Puerto Rico (Mercado‐Molina et al., 2015), and parts of Los Roques National Park, Venezuela (Croquer et al., 2016). Efforts at population restoration of *Acropora* have resulted in some local success (Schopmeyer et al., 2017), although many efforts were decimated by the 2023 marine heatwave (Cornwall, 2024; Thompson et al., 2025). Overall, *Acropora* spp. in the Caribbean now occurs at low abundances compared with 40 years ago. Given the spatial scale over which their population densities have been reduced (Jackson et al., 2014), regional isolation and lack of admixture between Eastern and Western Caribbean populations (Baums et al., 2005; García‐Urueña et al., 2022), and their ecological importance, their current status would appear to fit the scientific definition of *rare* (Gaston, 1997; Rabinowitz, 1981); this rarity has recently transitioned *Acropora* spp. to functional extinction in Florida's Coral Reef (Manzello et al., 2025).

## IMPLICATIONS OF RARITY

Increased rarity threatens maintenance of the ecological functions of affected species; yet, descriptions of these effects require those functions to be defined. Functional traits influence species performance and can be used to predict population turnover, biomass, and their roles in ecosystem (Bellwood et al., 2018; Shipley et al., 2006; Violle et al., 2007). Traits conferring strong competitive abilities play a greater role in determining population persistence than local abundances (Rabinowitz, 1981), and colony growth form can be a predictor of fitness (McWilliam et al., 2023). Tolerance of extreme environmental conditions, such as those involving temperature, turbidity, and salinity, and traits that confer reproductive success may be important drivers of coral rarity (DeVantier & Turak, 2017; Foden et al., 2013). Colony size is positively associated with fecundity (Alvarez‐Noriega et al., 2016), and size‐specific fecundity is higher in common versus uncommon corals (McWilliam et al., 2023). Although coral trait values (e.g., Madin et al., 2016) can reveal physiological and competitive functions, many of the appropriate traits have not been measured, and where data are available, high trait values have an equivocal connection to fitness. Arguably of greater importance than impaired functionality is the risk of extinction, for widespread rarity may ensure extinction (Hull et al., 2015), and any large reduction in population sizes and colony fecundity is likely to reduce larval dispersal and the extent of outcrossing, leading to genetic bottlenecks and inbreeding (Hartfield, 2015).

## CHALLENGES IN SAMPLING RARE CORALS

A fundamental concern in the study of coral biology is the limited capacity for the taxonomic identification of corals in the field (Grupstra et al., 2024). Although conservation at the ecosystem scale is crucial to preserve ecological, environmental, and evolutionary processes (Kleypas et al., 2021; Mace, 2004), ecosystem conservation depends on the species that comprise it, and these processes can only be addressed if the species can be accurately identified. The genomics revolution has challenged many of the species’ concepts for scleractinian corals that relied on corallum morphology (Fukami et al., 2008; Stanton et al., 2019), and so abundance assessments of rare corals can now benefit from emerging taxonomic resolution. Improvements in the capacity to identify species usually lead to increases in the number of species resolved (Mace, 2004), although each species is then characterized by a smaller population size and often a more restricted range. Although we suggest that there is an immediate and critical need to better resolve coral species in situ, we suspect that it is unrealistic to expect a single solution to quickly emerge to meet the need of identifying coral species to advance efforts for coral conservation (Wheeler, 2004). Some applications will demand the highest resolution of genetic identification, some will advance with morphologically defined species, and some will rely on operational taxonomic units (OTUs). We advocate for not letting perfection be the enemy of progress. The need to study rare corals requires immediate action, even if genetic identification requires subsequent revisions of sampling, interpretations, and estimates of population size.

For *Dendrogyra* and *Ctenella*, taxonomic identification is made easier by the inferred single species in each genus, but the screening of other species for rarity promises to be challenging when cryptic species occur (Hernández‐Agreda et al., 2024; Matias et al., 2023), or species complexes comprising multiple hybrids (syngameons) arise (Richards & Hobbs, 2015; Richards et al., 2008). Although extinction is almost universally understood as the complete loss of a species (Hughes et al., 2021), its empirical definition is debated. Extinction was often assumed when a species had not been seen for 50 years (Groombridge et al., 1994), but this has been updated to species for which “there is no reasonable doubt that the last individual has died” (Smith & Solow, 2012) and that have satisfied the criteria defined by extinction probability models (Fisher & Humphreys, 2024).

Much of coral reef ecology relies on surveys in which spatially limited techniques, such as quadrats, video transects, and line‐intercept transects, were used (Colwell & Coddington, 1994; Loya & Slobodkin, 1971). In these cases, there are at least 2 factors that might affect measures of abundance for rare taxa. First, as survey area increases, the spatial heterogeneity of species distribution becomes apparent. Second, as the number of survey replicates is increased, so does the likelihood of detecting rare species (Smith & Solow, 2012). With some corals now occurring at such low densities that none are found on a single survey, zero‐inflated values are frequently returned from sampling replicates (e.g., 1 × 1‐m quadrats) mismatched to the scale of distribution of the coral or corals being surveyed. For reef corals, low colony abundances may require quantification on a hectare scale (or larger), for example, through georeferenced surveys (Miller et al., 2023) or aerial mapping (Asner et al., 2020). Sampling must also address the distributional range of the selected species, which requires surveys over a landscape scale (i.e., 20–200 km [Mittelbach et al., 2001]).

Recent technological advances have the potential to revolutionize surveys of coral reefs at large spatial scales. Large‐area imaging offers the potential for high‐resolution analysis of coral communities (Edwards et al., 2017), although the ultimate efficacy of this approach will depend on the visual acuity of the images (pixel resolution relative to the features used to identify the corals) and the ability to resolve species by morphology. Citizen scientists trained to recognize morphologically unique coral species (e.g., Figure 1) can effectively survey large areas of reef, unless the corals are too small to be detected or cannot be reliably identified by morphology. More recently, autonomous or semiautonomous video platforms have advanced to a stage where they offer promise in searching coral reefs for rare corals (Girdhar et al., 2023). With sufficient optical resolution, including the capacity for high‐resolution 3‐dimensional reconstructions, the images from such systems can be analyzed with automated tools, such as CoralNET software (Beijbom et al., 2015), that can identify corals to genus. To date, the ultimate taxonomic resolution of these approaches remains unknown, but the rapid development of imaging technology and the capacity for artificial intelligence (AI) to identify objects provide optimism that the full capacity of these systems has not been realized. With accurate georeferencing (Miller et al., 2023), images can be used to spatially locate rare corals for subsequent visits and genetic sampling. Advances in next‐generation sequencing and its application to environmental DNA (eDNA) and metabarcoding of marine communities offer a potential means for species‐specific biomonitoring (Shelton et al., 2016; Apprill et al., 2023). This technique has already been used to detect coral spawning from seawater samples collected from above the spawning corals (Ip et al., 2023) and to estimate coral cover (Nichols & Marko, 2019).

Regardless of the technique employed, traditional and innovative techniques return estimates of coral population density that can be used to evaluate the current population status without quantifying their dynamics; analysis of coral population dynamics requires repeated surveys of multiple colonies of each species (Edmunds & Riegl, 2020). The population dynamics of rare corals are particularly important because they inform discussions of their likely fate through population viability analysis (PVA), which includes accurately quantifying the risks of extinction (Coulson et al., 2001). To our knowledge, a PVA has only been completed for *Seriatopora hystrix* (Muko et al., 2014).

## RESEARCH NEEDS

Studying rare corals is predicated on being able to accurately identify them to species underwater. Appropriate plans for species conservation require quantification of their distribution (Riginos & Beger, 2022) and accurate taxonomic identification, which in turn is required to evaluate their contribution to ecosystem function (Winfree et al., 2015). The data required to conduct definitive assessments of rarity do not currently exist for the vast majority of coral species. Advances in genomics have meant that the historic taxonomy of numerous contemporary coral species has been challenged (Bridge et al., 2023; Huang et al., 2011; Schmidt‐Roach et al., 2013). Modern taxonomic revisions of corals aid in delineating species composed of multiple genetically distinct taxa, which is information that is critical for the conservation of clusters of individuals that may be interbreeding (Hey et al., 2003). The ongoing development of rapid in situ genetic testing (e.g., using the MinION portable DNA sequencer [Menegon et al., 2017]) is likely to be a transformative advance for the study of rare corals.

**FIGURE 2:**
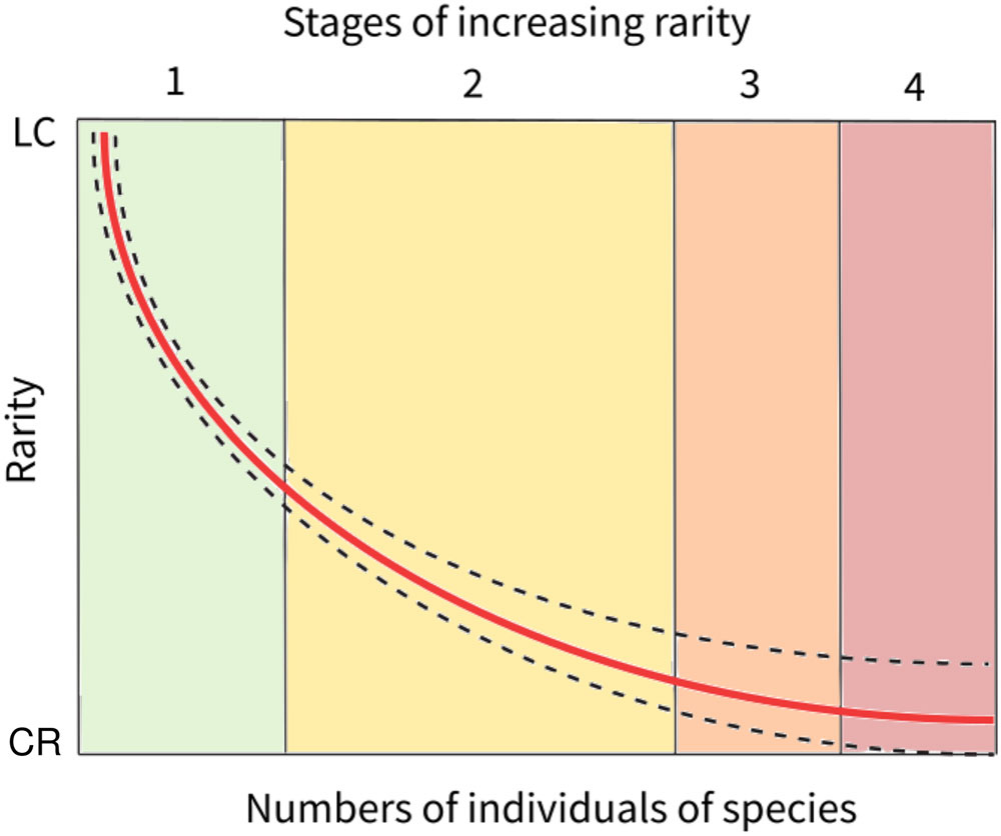
Relationship between the 4 stages of rarity (International Union for Conservation of Nature categories least concern [LC] to critically endangered [CR]) and the number of individuals of a species for a given species (red line) (dashed lines, error of density measures; bars, rarity increases from green to pink and is based on observation). Rarity is assessed by in situ observations matched to subsequent ecological approaches to population monitoring and management.

The rapid changes that have affected coral reefs in the last several decades have changed research priorities in the field (Bellwood et al., 2019; Fisher et al., 2011). For benthic communities, attention has remained focused on stony corals (scleractinians, milleporine hydrocorals, and tubiporid octocorals) because they are the ecosystem engineers of coral reefs. With worldwide local population depletion and functional extinction of stony corals, regional‐scale changes in species assemblages and mass rarity may pose more immediate threats than imminent global extinction for the Scleractinia (Dietzel et al., 2021). We emphasize aspects of these trends and corresponding research actions in Table 1 and Figure 2, and we identified 2 themes for much needed future research: accurate identification and quantification of rare corals and the animals, algae, and microbes with which they are associated and characterization of rare corals.

**TABLE 1:** Assessment of rarity (corresponding to International Union for Conservation of Nature categories least concern [LC] to critically endangered [CR]) by in situ observations matched to subsequent ecological approaches to population monitoring and management.

Rarity^a^	Approach	In situ observation
1 Least concern to near threatened	Traditional benthic surveys	Rapidly declining abundance, an early warning
2 Vulnerable	Revised sampling methods, e.g., greater replication, increased frequency, larger quadrats	Dramatic reduction in abundance
3 Endangered	Radically different sampling methods, e.g., population viability analysis (PVA), genetic sampling, niche evaluation	Persistent low abundance
4 Critically endangered	Proactive conservation measures, e.g., movement of colonies into captivity, translocation of colonies, biobanking	Rare, leading to extinction without intervention

^a^ Numbers correspond to colored bars in Figure 2.

### Accurate identification and quantification

When corals occur at low population densities, quantification of population sizes with quadrat, transect, plot, and line‐intercept transects is inefficient and prone to low accuracy in determining organism abundances. Effective studies of rare corals will remain unobtainable until coral species can be reliably identified, most likely through genetic tools. The sampling challenges of enumerating rare corals will require greater use of large‐area sampling by all means available (e.g., roving surveys by divers and snorkelers, large image sampling, or automated image‐based surveys). Some of the promising approaches are likely to involve georeferencing coral colonies through remote sensing, autonomous underwater vehicles (AUVs), remotely operated vehicles (ROVs), eDNA, and emerging technologies that combine multiple approaches guided by AI and machine learning in a single platform (Apprill et al., 2023).

Corals often host a complex community of symbionts, including microbes and dinoflagellate algae (i.e., the holobiont [Rohwer et al., 2002]), as well as vertebrates and invertebrates (Hoeksema et al., 2012). Taxa inhabiting endangered corals, especially those that are obligate associates, also have a high extinction risk (Säterberg et al., 2013; van der Schoot & Hoeksema, 2024). Studies of the biology and ecology of rare coral should encompass the holobiont and the cryptic communities with which they are associated.

### Characterization of rare corals

Before the population biology of accurately identified rare corals can be quantified, benchmarks for population density and geographic range will need to be established in order to accurately and repeatably codify rare corals. Guidance in defining rarity is in the scientific literature on terrestrial and fossil domains and from the IUCN, but the study of rare corals can begin only with rigorously defining the concept. A secondary concern is that the quantification of the characteristics of rare corals is data intensive, and such efforts have remained uncommon for slow‐growing and long‐lived species (Caswell, 2000). Biological fields other than marine biology and coral reef science have advanced these topics, and the literature (e.g., Kunin & Gaston, 1997; Soule, 1986) provides examples of topics pertaining to determining whether select coral species are adapted to rarity, evaluating which life‐history traits have allowed habitually uncommon corals to persist through evolutionary time, and determining the degree of rarity that leads to extinction.

Our intent is to draw attention to one of the critical facts currently receiving insufficient focus in coral biology: many corals are becoming rare. However, this article is not intended to provide an exhaustive treatment of the topic, nor do we imply that there are answers to many of the topics we addressed. Instead, we highlight this overlooked aspect of the coral reef crisis in the hope that the direst outcome of declining abundance trends—extinction—can be avoided. Applying historic, contemporary, and novel approaches to the study and conservation of rare (and likely endangered) coral species has never been more urgent.
